# Resveratrol Protects Against Post-Contrast Acute Kidney Injury in Rabbits With Diabetic Nephropathy

**DOI:** 10.3389/fphar.2019.00833

**Published:** 2019-07-26

**Authors:** Yongfang Wang, Bin Wang, Xun Qi, Xin Zhang, Ke Ren

**Affiliations:** ^1^Department of Radiology, First Hospital of Shanxi Medical University, Taiyuan, China; ^2^Department of Radiology, First Hospital of China Medical University, Shenyang, China; ^3^Department of Radiology, Xiang’ an Hospital of Xiamen University, School of Medicine, Xiamen University, Xiamen, China

**Keywords:** PC–AKI, diabetic nephropathy, SIRT1, PGC-1α, HIF-1α, apoptosis

## Abstract

Resveratrol (Res) is a multi-functional polyphenol compound that has protective functions in acute kidney diseases. Here, we examined whether the resveratrol could ameliorate post-contrast acute kidney injury (PC–AKI) following diabetic nephropathy (DN), and explored any underlying mechanism(s) *in vivo* and *in vitro*. Twenty-four rabbits with DN were randomly divided into four groups: control (Cont), resveratrol (Res), iohexol (PC–AKI), and resveratrol plus iohexol (Res+PC–AKI) groups. Functional magnetic resonance imaging, renal histology, blood and urinary biomarkers, silent information regulator l (SIRT1), peroxisome proliferator-activated receptor gamma coactivator-1 alpha (PGC-1α), hypoxia-inducible transcription factor-1α (HIF-1α), and apoptosis-associated protein expression were assessed *ex vivo*. For *in vitro* experiments, renal tubular epithelial (HK-2) cells subjected to high glucose conditions were treated with resveratrol, Ex527, an SIRT1 inhibitor, or 2-methoxyestradiol (2-MeOE2), HIF-1α inhibitor, before treatment with iohexol. With regard to the rabbit model of acute renal injury in DN, compared to the PC–AKI group, the Res+PC–AKI group showed decreased levels of cystatin C and urinary neutrophil gelatinase-associated lipocalin, increased pure molecular diffusion (*D*) and the fraction of water flowing in capillaries (*f*), a decreased apparent relaxation rate (R2*), renal injury score and apoptosis rate, increased protein expression levels of SIRT1 and PGC-1α, and decreased levels of HIF-1α and apoptosis-associated protein. In addition, iohexol decreased HK-2 cell survival and increased the cell apoptosis rate; results were reversed after treating cells with resveratrol. Resveratrol reduced renal hypoxia, mitochondrial dysfunction and renal tubular cell apoptosis by activating SIRT1–PGC–1α–HIF-1α signaling pathways in PC–AKI with DN.

## Introduction

Post-contrast acute kidney injury (PC–AKI), described as a decrease in renal function that follows the intravascular administration of contrast medium, is one of the leading causes of hospital-acquired acute kidney injury (Takahashi et al., 2017; Van Der Molen et al., 2018). This type of injury is associated with adverse short- and long-term outcomes (Maioli et al., 2012; Chalikias et al., 2016). Overwhelming epidemiological and clinical evidence has demonstrated that diabetes mellitus may significantly contribute to the development of PC–AKI after the application of contrast medium (Tziakas et al., 2014; Chalikias et al., 2016). Therefore, the identification of new molecular target(s) has been a major focus for the prevention of PC–AKI in high-risk patients.

Silent information regulator l (SIRT1) is a nicotinamide adenine dinucleotide-dependent protein deacetylase that is the human homolog of yeast silent information regulator 2 (Xu et al., 2016; Morigi et al., 2018). Resveratrol, as a chemical SIRT1 activator, can effectively repair SIRT1 activity. Accumulated experimental evidence suggests that resveratrol may inhibit the inflammatory response and tubular apoptosis, and scavenge oxygen free radicals in cisplatin-induced nephropathy, renal ischemic/reperfusion injury and diabetic nephropathy (Kim et al., 2011; Gu et al., 2016; Xiao et al., 2016).

Recent studies have shown that resveratrol promotes the activation of peroxisome proliferator-activated receptor-gamma coactivator-1 alpha (PGC-1α), and decreases oxidative stress and apoptosis in acute renal injury. PGC-1α is a nuclear-encoded transcriptional coactivator that regulates the expression of nuclear-encoded mitochondrial proteins (Ishimoto and Inagi, 2016). As reported, the overexpression of PGC-1α produces a robust increase in mitochondrial numbers, respiratory capacity and intracellular ATP concentration in cultured proximal tubular cells, and may promote repair and recovery after hypoxia damage (Rasbach and Schnellmann, 2007). Previous studies have shown that hypoxia-inducible transcription factors (HIFs), recognized as master regulators of hypoxic adaptation, are detected during the development of PC–AKI (Rosenberger et al., 2005). Many molecular pathways are involved in the protective effects of resveratrol, but the role of the SIRT1–PGC–1α–HIF-1α pathway in PC–AKI–based diabetic nephropathy has not yet been fully described. The aim of the present study was to assess the effect of resveratrol on the development of PC–AKI in diabetic models and to provide experimental evidence for the prevention and treatment of PC–AKI.

## Materials and Methods

### Animals and Experimental Design

A total of 24 male white New Zealand rabbits (weighing 2.5–3.0 kg) were housed in an environment with a temperature of 22 ± 1°C, relative humidity of 50 ± 1% and a light/dark cycle of 12/12 h. All animal studies (including rabbit euthanasia) were conducted according to the China Medical University Application for Laboratory Animal Welfare and Ethical Review (201802 Edition). The protocol was approved by the Ethics Review Committee of China Medical University.

All animals were acclimatized for 7 days before the start of each experiment. Rabbits with diabetic nephropathy (DN) and renal impairment were established by a single intravenous injection of alloxan monohydrate (100 mg/kg) dissolved in 0.9% saline after 12 h of overnight fasting (Zhao et al., 2016). Furthermore, the rabbits were treated with 10% glucose solution for 24 h to get through the early stage of drug-induced hypoglycemia. Three days after alloxan injection, blood was collected and fasting blood glucose levels analyzed. A successful diabetes model was established when glucose levels were > 16.7 mmol/L (Zhao et al., 2016). Consequently, blood glucose levels were monitored once a week.

Twelve weeks after the induction of diabetic nephropathy (Wang et al., 2014), rabbits were randomly divided into four groups: saline–treated control group (Cont; *n* = 6), resveratrol alone–treated group (Res; *n* = 6), contrast medium–treated group (PC–AKI; *n* = 6), and contrast medium plus resveratrol–treated group (PC–AKI + Res; *n* = 6; **Figure S1**). The animals were treated with or without resveratrol (80 mg/kg/d, p.o.; Sigma, St Louis, MI, USA) once a day, for a total of 14 days before applying iohexol, a contrast agent (Li et al., 2016). Furthermore, iohexol (Omnipaque; 350 mg iodine/ml, 830 mOsm/kg H_2_O; GE Healthcare, Shanghai, China) was intravenously injected at a dose of 2.5 g iodine/kg body weight *via* a venous cannula (24 G) inserted in the marginal ear vein (Bhargava et al., 1990; Lauver et al., 2014).

### Functional MRI Mapping and Data Analysis

Animals underwent magnetic resonance imaging (MRI) 24 h after the injection of iohexol. MR imaging was performed on a 3.0-T scanner (General Electric Medical Systems, Milwaukee, WI, USA) using a cardiac matrix coil. An advanced workstation (General Electric Medical Systems) was used to extract intravoxel incoherent motion (IVIM) and blood oxygenation level–dependent (BOLD) parameters, including *D*, D*, *f* and R2* values. Quantitative regional IVIM parameters and R2* measurements were performed, using manually defined region-of-interest (ROI) measurements, by two experienced radiologists who remained blinded to each group assignment. Regions of interest were manually placed in the renal cortex (CO) and outer medulla (OM; **Figure S2**) according to a previously described approach (Prasad et al., 2010).

IVIM parameters were consecutively calculated using a nonlinear biexponential fit based on the following equation: *S* *_b_*/*S* *_0_* = (1-*f*). exp (-*b* *D*) + *f*. exp [-*b* (*D* + D*)], where *S* *_0_* = signal intensity in the absence of the diffusion weighting (*b* = 0); *S* *_b_* = signal intensity with diffusion gradient *b*; slow diffusion coefficient (*D*) = pure molecular diffusion; fast diffusion coefficient (D*) = flow velocity; and the perfusion fraction (*f*) = the fraction of water flowing in capillaries (Hu et al., 2015). BOLD parameters were able to indirectly measure the partial oxygen pressure by estimating the deoxyhemoglobin concentration in the renal parenchyma and blood vessels (Liu et al., 2013). Data acquisition of IVIM and BOLD parameters is shown in **Table 1**.

**Table 1:** MRI parameters used for IVIM and BOLD parameters.

	IVIM	BOLD
Sequence type Orientation Repetition time, ms	DW–EPI Coronal 4,050	2D–GRE Coronal 96.2
Echo time, ms	95.5	4.4–48.7
Flip angle	–	30°
Bandwidth, hertz per pixel	–	41.67
Field of view, cm^2^	16.0 × 16.0	16.0 × 16.0
Matrix Section thickness, mm	160 × 160 3.0	256 × 256 3.0
Number of excitations	2.0	2.0
Acquisition time	6 min 41s	4 min 10 s
*b*-values (s/mm^2^)	0, 10, 20, 50, 100, 200, 400, 600, 800, 1,200	–

MRI, magnetic resonance imaging; IVIM, intravoxel incoherent motion; BOLD, blood oxygenation level–dependent; DW–EPI, diffusion-weighted echo-planar imaging; 2D-GRE, two-dimensional gradient-recalled echo.

### Renal Function Biomarkers

A total of 1.0–1.5 ml of blood and urine samples was collected from each animal after MRI. Blood samples were centrifuged and used to examine serum creatinine (Cr), blood urea nitrogen (BUN), and cystatin C (CysC) levels. Urine samples were collected for the assessment of urine creatinine and urinary neutrophil gelatinase-associated lipocalin (uNGAL). Concentrations of CysC and NGAL were assessed using an enzyme-linked immunosorbent assay (ELISA) kit (Yuanmu Biological Technology Co., Ltd, Shanghai, China) according to the manufacturer’s instructions, and concentration levels of NGAL were normalized to the urine creatinine concentration (Li et al., 2014a; Li et al., 2014b). Serum Cr, BUN, and urine Cr were analyzed by a local clinical laboratory.

### Histological Observations

After MRI, rabbits were sacrificed, and the right kidneys removed and fixed in 4% buffered paraformaldehyde for 72 h. Samples were then dehydrated, embedded in paraffin, cut into 5 μm sections, and finally stained with hematoxylin and eosin (H&E). Histological assessment of renal damage included observing tubular vacuolization and degeneration, renal tubular casts, inflammatory infiltration and obvious necrosis in the cortex, and the disruption of the tubular structures in the outer medulla after iohexol administration. Renal alterations were graded as follows: 0, normal kidney; 1, minimal injury (0–5%); 2, moderate injury (5–25%); 3, intermediate injury (25–75%), and 4, severe injury (75–100%) (Colbay et al., 2010). Two independent pathologists, who were blinded to the experimental conditions, evaluated the samples according to the scoring criteria. Renal cell apoptosis was assessed using terminal deoxynucleotidyl transferase dUTP nick-end labeling (TUNEL) staining (WL029a; Wanleibio, Shenyang, China) as previously described (Deng et al., 2015) and the percentage of apoptotic cells was quantified using ImageJ software.

### *In Vitro* Experiments

Immortalized human proximal tubule (HK-2) cells were purchased from Basic Medical Cell Center of School of Basic Medicine, Peking Union Medical College (Beijing, China). Cells were grown in low-glucose (LG, 5.5 mM) RPMI 1640 medium (Gibco Laboratories, Grand Island, NY, USA) containing 10% fetal calf serum (Gibco Laboratories) and cultured in a humidified atmosphere containing 5% CO_2_/95% air at 37°C. The culture medium was supplemented with a high level of glucose (HG, 25 mM) to induce a DN cell model. An *in vitro* model of contrast injury was constructed based on a previously described method (Billings et al., 2008). The SIRT1 inhibitor, Ex527, and HIF-1α inhibitor, 2-methoxyestradiol (2-MeOE2), were purchased from Selleckchem (Shanghai, China), while the SIRT1 activator, resveratrol, was obtained from Abcam (Cambridge, UK).

Well-differentiated HK-2 cells were divided into five groups: high glucose + resveratrol + iohexol (25 mmol/L glucose + 50 μmol/L Res + 100 mg/ml iohexol; Res), high glucose + Ex527 + iohexol (25 mmol/L glucose + 10 μmol/L Ex527 +100 mg/ml iohexol; Ex527), high glucose + iohexol (25 mmol/L glucose + 100 mg/ml iohexol; PC–AKI), high glucose + resveratrol + 2-MeOE2 + iohexol (25 mmol/L glucose + 50 μmol/L Res + 10 μmol/L 2-MeOE2 +100 mg/ml iohexol; 2-MeOE2), and low glucose (5.5 mmol/L glucose; Cont; **Figure S3**). The cells were stimulated under high glucose conditions for 24 h (Chen et al., 2018). In order to evaluate the effect of activating SIRT1, the cells were pretreated with Ex527 for 24 h or 2-MeOE2 for 2 h before the iohexol treatment. After Res, Ex527 or 2-MeOE2 treatment, cells were exposed to iohexol for 16 h.

### CCK-8 Cytotoxic Assay

In order to evaluate the protective effect of resveratrol, HK-2 cell viability in the five groups was quantified by a Cell Counting Kit-8 (CCK-8) assay (Dojindo Laboratories, Shanghai, China). Briefly, cells were incubated with 10 µl CCK-8 test solution for 2 h at 37°C. Absorbance values were measured at 450 nm using a microplate reader (Thermo Fisher Scientific, Waltham, MA, USA).

### Flow Cytometry Analysis of Cell Apoptosis

HK-2 cells were seeded into 6-well plates at a density of 1 × 10^5^ cells/well. The treated HK-2 cells were harvested and stained using an Annexin V- Light 650 Apoptosis Detection Kit (Wanleibio; WLA002b, Shenyang, China). After incubation for 15 min in the dark, the cell apoptosis ratio was measured using a flow cytometer (Acea Bio, San Diego, CA, USA).

### Western Blot

Both kidney tissue and isolated HK-2 cells were centrifuged at 12,000 rpm for 20 min after homogenization in radio-immunoprecipitation assay lysis buffer containing a protease inhibitor cocktail. Total protein concentrations were determined by a bicinchoninic acid method. Afterward, protein samples were boiled at 95°C for 5 min. About 50 µg equal amounts of protein samples were separated by gel electrophoresis on an 8% to 12% sodium dodecyl sulfate–polyacrylamide gel and then transferred onto a polyvinylidene diﬂuoride membrane using wet transfer at 80 V for 60 min, and 120 V for 60 min. Nonspecific binding sites were blocked with 5% skim milk powder at room temperature for 2 h and the membrane then incubated with the following primary antibodies: SIRT1 (1:500, abs120289; absin, Shanghai, China), PGC-1α (1:500, TA343708; OriGene, Rockville, MD, USA), HIF-1α (1:500, NB100-105; Novus Biologicals, Centennial, CO, USA), Bax (1:5000, 50599-2-Ig; ProteinTech, Wuhan, China), Bcl-2 (1:2000, 12789-1-AP; ProteinTech, Wuhan, China), cleaved caspase-3 (1:1,000, ab2302; Abcam, UK), and cytochrome C (Cyt-C; 1:5,000, NB100-56503; Novus, Saint Charles, MI, USA) at 4°C overnight. After washing, films were incubated with horseradish peroxidase-labeled conjugated secondary antibodies (Proteintech) for 2 h. The immunostained protein bands were visualized with an enhanced chemiluminescence system and analyzed using Image J software. Beta-actin was used as an internal control.

### Statistical Analysis

All values were analyzed with SPSS22.0 (SPSS Inc., Chicago, IL, USA) or GraphPad Prism 5 and expressed as mean ± standard deviation. Continuous data were analyzed with a one-way analysis of variance (ANOVA) followed by Fisher’s least significance difference test between multiple groups, and unpaired data were analyzed with the Kruskal–Wallis test. The Mann–Whitney test was used for nonparametric comparisons between the two groups. *P* < 0.05 was considered statistically significant.

## Results

### Effects of Resveratrol on Renal Function

**Figure 1:**
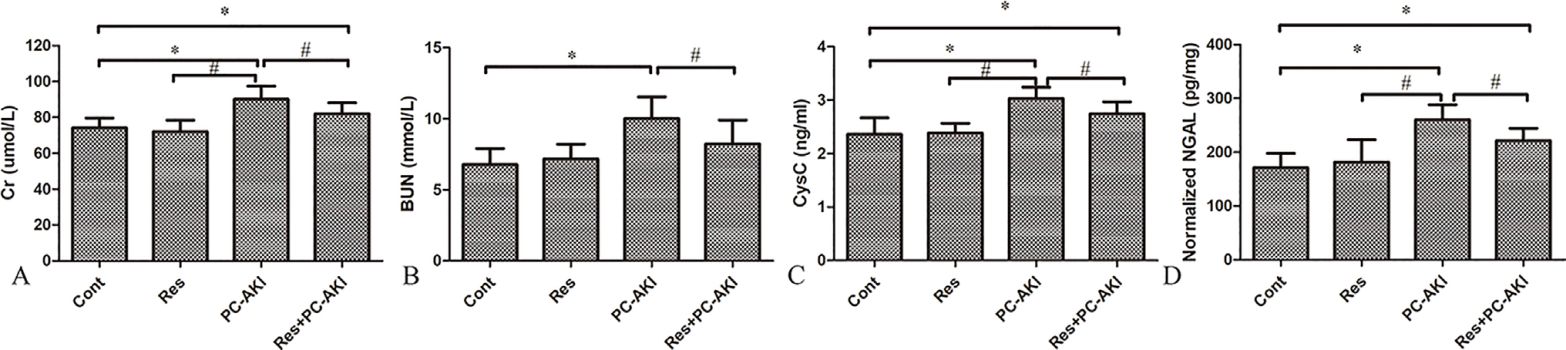
Changes in renal function were evaluated after the injection of iohexol and co-administration of resveratrol in rabbits. Iohexol caused renal dysfunction in the PC–AKI group, while the co-administration of resveratrol decreased the level of Cr **(A)**, BUN **(B)**, CysC **(C)** and NGAL **(D)**. *P < 0.05 vs. Cont; ^#^ *P* < 0.05 vs. PC–AKI. Cr, serum creatinine; BUN, urea nitrogen; CysC, cystatin C; NGAL, neutrophil gelatinase–associated lipocalin; Cont, control; Res, resveratrol; PC–AKI, post-contrast acute kidney injury.

Contrast medium significantly increased serum Cr, BUN, and CysC levels and the uNGAL level. In contrast, serum Cr, BUN and CysC levels were significantly lower in the Res+PC–AKI group compared to the PC–AKI group (*P* = 0.038; *P* = 0.035; *P* = 0.048, respectively), whereas there was no significant difference between Cont and Res groups (*P* = 0.562; *P* = 0.631; *P* = 0.895, respectively). Furthermore, compared with the Cont group, the urinary concentrations of NGAL were significantly increased 24 h after iohexol injection in the PC–AKI group (*P* < 0.001); however, resveratrol treatment markedly reduced levels of uNGAL induced by iohexol in the Res+PC–AKI group (*P* = 0.04; **Figure 1**). Thus, resveratrol ameliorated acute renal dysfunction after exposure to contrast medium in DN.

### Effect of Resveratrol on Functional MRI Parameters

**Figure 2:**
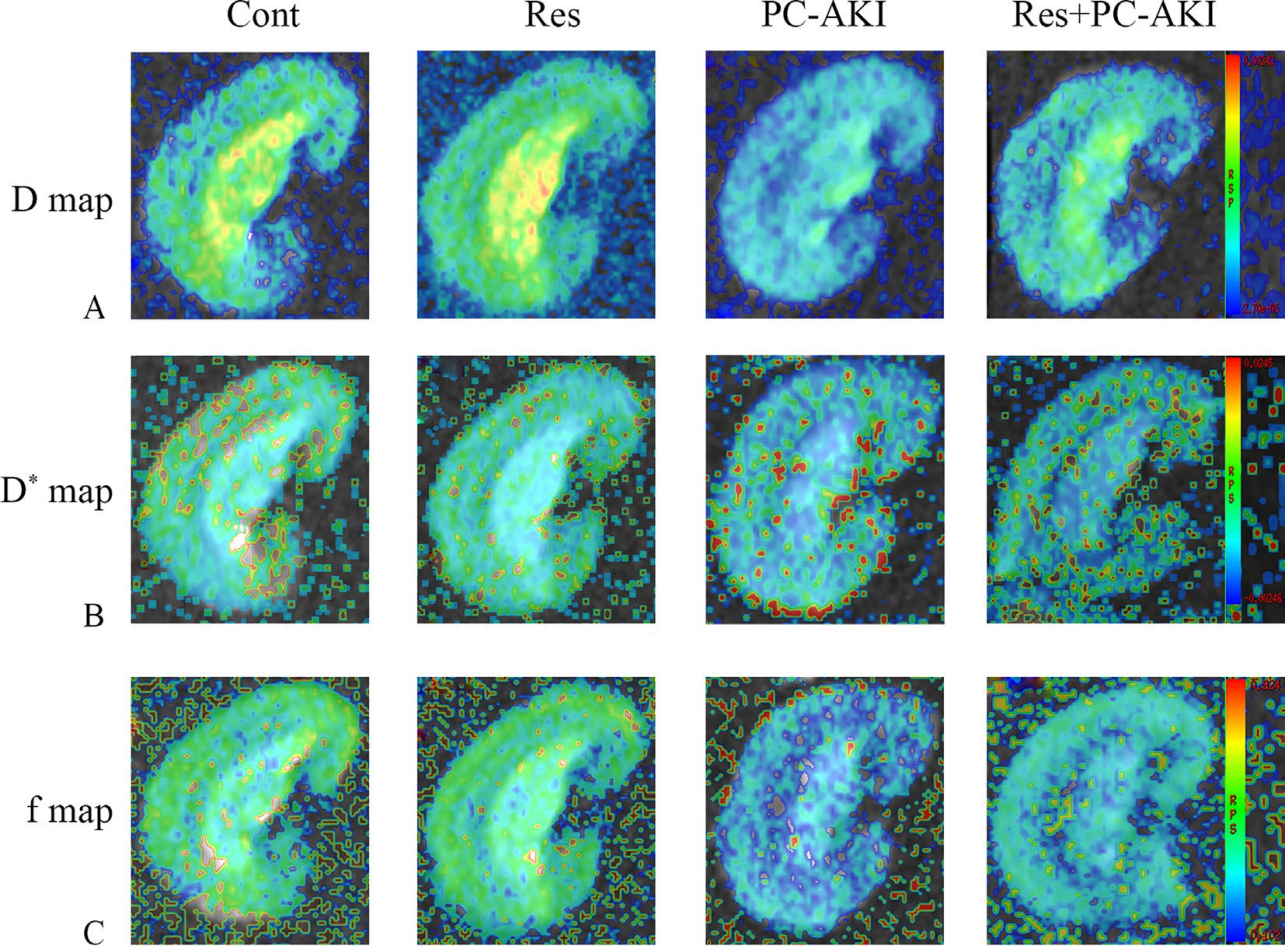
Parametric images obtained from the intravoxel incoherent motion (IVIM) model for rabbit with different treatments. Representative IVIM images of *D* **(A)**, D* **(B)**, and *f* **(C)** maps. The maximum *D*, D* and *f* signal changes were observed in the PC–AKI group. The *D*, D* and *f* values increased after the application of resveratrol. Cont, control; Res, resveratrol; PC–AKI, post-contrast acute kidney injury; IVIM, intravoxel incoherent motion.

**Figure 3:**
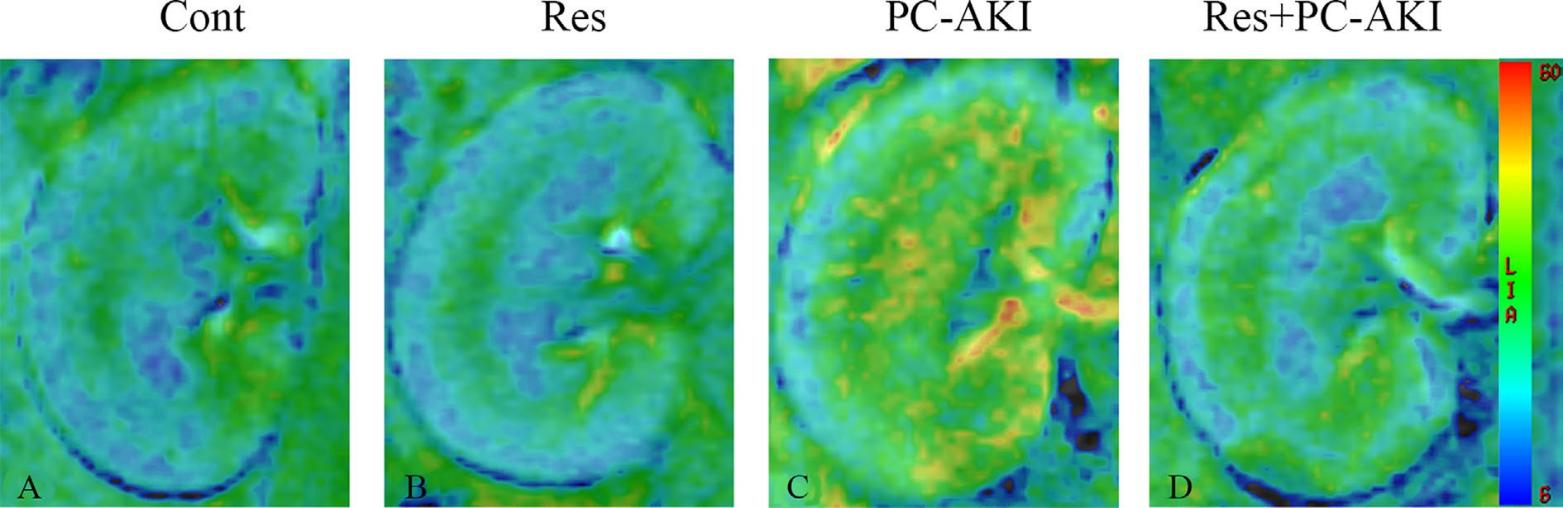
Parametric images obtained from the BOLD model for rabbits with different treatments. The window and level settings for each map were kept the same. Compared to the control group, the brighter R2* maps in the PC–AKI group indicated increased hypoxia after iohexol injection. In the Res+PC–AKI group, the R2* values were significantly lower compared to the PC–AKI group, indicating increased oxygenation when using resveratrol before iohexol. **(A)** Control group, **(B)** Res group, **(C)** PC–AKI group, **(D)** Res+PC–AKI group. Cont, control; Res, resveratrol; PC–AKI, post-contrast acute kidney injury; BOLD, blood oxygenation level–dependent.

The IVIM parameters and R2* values between the four groups of rabbits are presented in **Table 2** and their corresponding representative images are shown in **Figures 2**, **3**.

**Table 2:** Summary of IVIM parameters and R2* for kidneys of rabbits with different treatments.

Kidney tissue	Groups	Cont	Res	PC–AKI	Res+PC–AKI
CO	D (×10^-4^ mm^2^/s)	3.99 ± 0.28	4.02 ± 0.30^#^	2.93 ± 0.33*	3.58 ± 0.34*^#^
	D* (×10*^-3^* *mm* *^2^* */s*)	9.86 ± 0.28	9.86 ± 0.30^#^	9.31 ± 0.35*	9.61 ± 0.38
	f (%)	39.13 ± 1.84	39.78 ± 1.90^#^	31.83 ± 1.67*	35.07 ± 2.91*^#^
	R2* (s^-1^)	23.42 ± 2.38	23.91 ± 4.92^#^	31.22 ± 3.07*	27.14 ± 1.58^#^
OM	D (×10*^-4^* *mm* *^2^* */s*)	3.74 ± 0.21	3.65 ± 0.42^#^	2.43 ± 0.28*	3.06 ± 0.46*^#^
	D* (×10*^-3^* *mm* *^2^* */s*)	8.97 ± 0.25	9.06 ± 0.27^#^	8.31 ± 0.32*	8.54 ± 0.42*
	f (%)	36.78 ± 1.78	37.20 ± 2.83^#^	28.75 ± 1.91*	33.28 ± 2.59*^#^
	R2* (s^-1^)	29.26 ± 4.85	28.65 ± 4.41^#^	41.32 ± 5.20*	35.40 ± 2.57*^#^

*P < 0.05 vs. Cont; ^#^P < 0.05 vs. PC–AKI.

Cont, control; PC–AKI, post-contrast acute kidney injury; Res, resveratrol; CO, renal cortex; OM, outer medulla.

After the injection of iohexol, *D* was significantly lower in the PC–AKI group compared with the Cont group in the CO (*P* < 0.0001) and OM (*P* < 0.0001). *D* was significantly higher in the Res+PC–AKI group compared with the PC–AKI group in the CO (*P* = 0.002) and OM (*P* = 0.009). Perfusion-related parameters of D* were slightly higher in the Res+PC–AKI group when compared with the PC–AKI group; however, significant differences were not found between the renal CO (*P* = 0.144) and OM (*P* = 0.214). In addition, the mean *f* values were significantly lower in the PC–AKI group compared to the Cont group in the CO (*P* < 0.0001) and OM (*P* < 0.0001). The mean *f* values significantly were greater in the Res+PC–AKI group compared with the PC–AKI group in the renal CO (*P* = 0.016) and OM (*P* = 0.003). The mean R2* values were significantly greater in the PC–AKI group compared with the Cont group in the renal CO (*P* < 0.0001) and OM (*P* < 0.0001), whereas R2* was lower in the Res+PC–AKI group when compared with the PC–AKI group in the renal CO (*P* = 0.041) and OM (*P* = 0.030). Thus, resveratrol increased oxygenation in kidney damage induced by exposure to contrast medium in DN.

### Effects of Resveratrol on Histopathological Changes and Renal Cell Apoptosis

**Figure 4:**
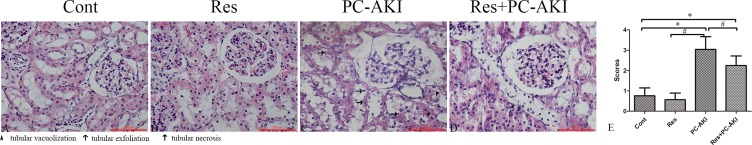
Representative photomicrographs of HE-stained kidney sections in rabbits with different treatments. Iohexol induced obvious morphological alterations in kidneys of rabbits in the PC–AKI group. Resveratrol reduced contrast medium–induced morphological changes in the Res+PC–AKI group. **(A)** Cont group, **(B)** Res group, **(C)** PC–AKI group, and **(D)** Res+PC–AKI group. **(E)** Renal injury scores. Original magnifications: ×400. *P < 0.05 vs. Cont; ^#^ *P* < 0.05 vs. PC–AKI. Cont, control; Res, resveratrol; PC–AKI, post-contrast acute kidney injury; HE, hematoxylin and eosin.

Histopathological examination showed no pathological alterations of the kidneys in the control group, whereas glomerular injury, tubular vacuolization–necrosis, and exfoliation of renal tubular epithelial cells, as well as the disruption of tubular structure scores were significantly greater in the PC–AKI group than in the other groups (**Figure 4**). Furthermore, the co-administration of resveratrol with iohexol (Res+PC–AKI) significantly decreased the severity score compared to the PC–AKI group (*P* = 0.015).

**Figure 5:**
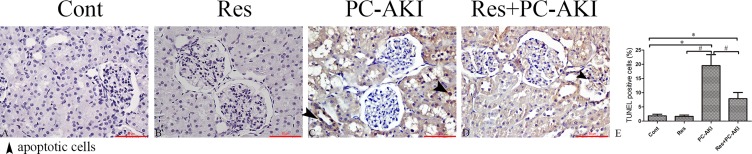
Representative images of transferase dUTP nick-end labeling (TUNEL) staining for rabbits with different treatments. **(A–D)** TUNEL staining **(E)** Quantitative analysis of TUNEL-positive cells. A significant increase in TUNEL-positive cells was observed in the kidneys of rabbits with DN 24 h after contrast medium injection, whereas resveratrol inhibited renal tubular cell apoptosis. Original magnifications: ×400. *P < 0.05 vs. Cont; ^#^*P* < 0.05 vs. PC–AKI. TUNEL, terminal deoxynucleotidyl transferase dUTP nick-end labeling; DN, diabetic nephropathy; Cont, control; Res, resveratrol; PC–AKI, post-contrast acute kidney injury.

As shown in **Figure 5**, the number of TUNEL-positive cells was increased in iohexol-treated kidneys, while resveratrol substantially decreased the number of apoptotic cells in the Res+PC–AKI group compared with that in the kidneys of the PC–AKI group (*P* = 0.001, *P* = 0.015). In short, resveratrol ameliorated pathological changes in the kidney in response to contrast medium exposure in DN.

### Effects of Resveratrol on Cell Toxicity Induced By Iohexol Under HG Conditions

**Figure 6:**
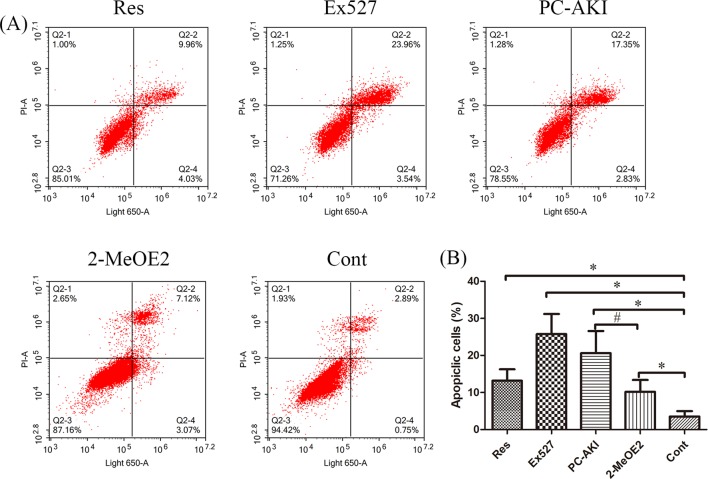
Quantitative analysis of HK-2 cell apoptosis by ﬂow cytometry. **(A)** HG-induced HK-2 cells were divided into five groups: Res, Ex527, PC–AKI, 2–MeOE2 and Cont groups. **(B)** Quantification of cell apoptosis in different groups. A significant increase in apoptotic cells was detected in the PC–AKI group; however, the up-regulation SIRT1 and silencing HIF-1α could both reduce iohexol-induced cell apoptosis. *P < 0.05 vs. Cont; ^#^ *P* < 0.05 vs. PC–AKI. HG, high glucose; PC–AKI, post-contrast acute kidney injury; Cont, control; Res, resveratrol; SIRT1, silent information regulator l; HIF-1α, hypoxia-inducible transcription factor-1α; Ex527, SIRT1 specific inhibitor; 2-MeOE2, HIF-1α specific inhibitor 2-methoxyestradiol; LG, low glucose. Groups: Res, HG+res+iohexol; Ex527, HG+Ex527+iohexol; PC–AKI, HG+iohexol; 2-MeOE2, HG+res+2-MeOE2+iohexol; Cont, LG.

As shown in **Table 3**, iohexol caused a marked elevation in HK-2 cell apoptosis when compared with controls (*P* = 0.005). In contrast, preincubation with resveratrol inhibited iohexol-mediated cell death (*P* = 0.196). Moreover, the HIF-1α inhibitor, 2-MeOE2, enhanced the renoprotective effect of resveratrol against cell apoptosis in iohexol-treated HK-2 cells under HG conditions (*P* = 0.047; **Figure 6**). In summary, resveratrol reduced cell cytotoxicity and apoptosis induced by contrast medium under HG conditions in a HIF-1α–mediated manner.

**Table 3:** Quantitative analysis of the percentage of viable cells in different groups.

Groups	Res	Ex527	PC–AKI	2-MeOE2	Cont
	0.5498 ± 0.1161	0.3419 ± 0.1448*	0.4093 ± 0.1825*	0.6334 ± 0.1885	1.0176 ± 0.1345

*P < 0.05 vs. Cont.

Cont, control; PC–AKI, post-contrast acute kidney injury; Res, resveratrol; Ex527, SIRT1 specific inhibitor; 2-MeOE2, HIF-1α specific inhibitor 2-methoxyestradiol; HG, high glucose; LG, low glucose.

Groups: Res, HG+res+iohexol; Ex527, HG+Ex527+iohexol; PC–AKI, HG+iohexol; 2-MeOE2, HG+res+2-MeOE2+iohexol; Cont, LG.

### Effects of Resveratrol on SIRT1–PGC-1α–HIF-1α Signaling in DN Rabbits *In Vivo*

**Figure 7:**
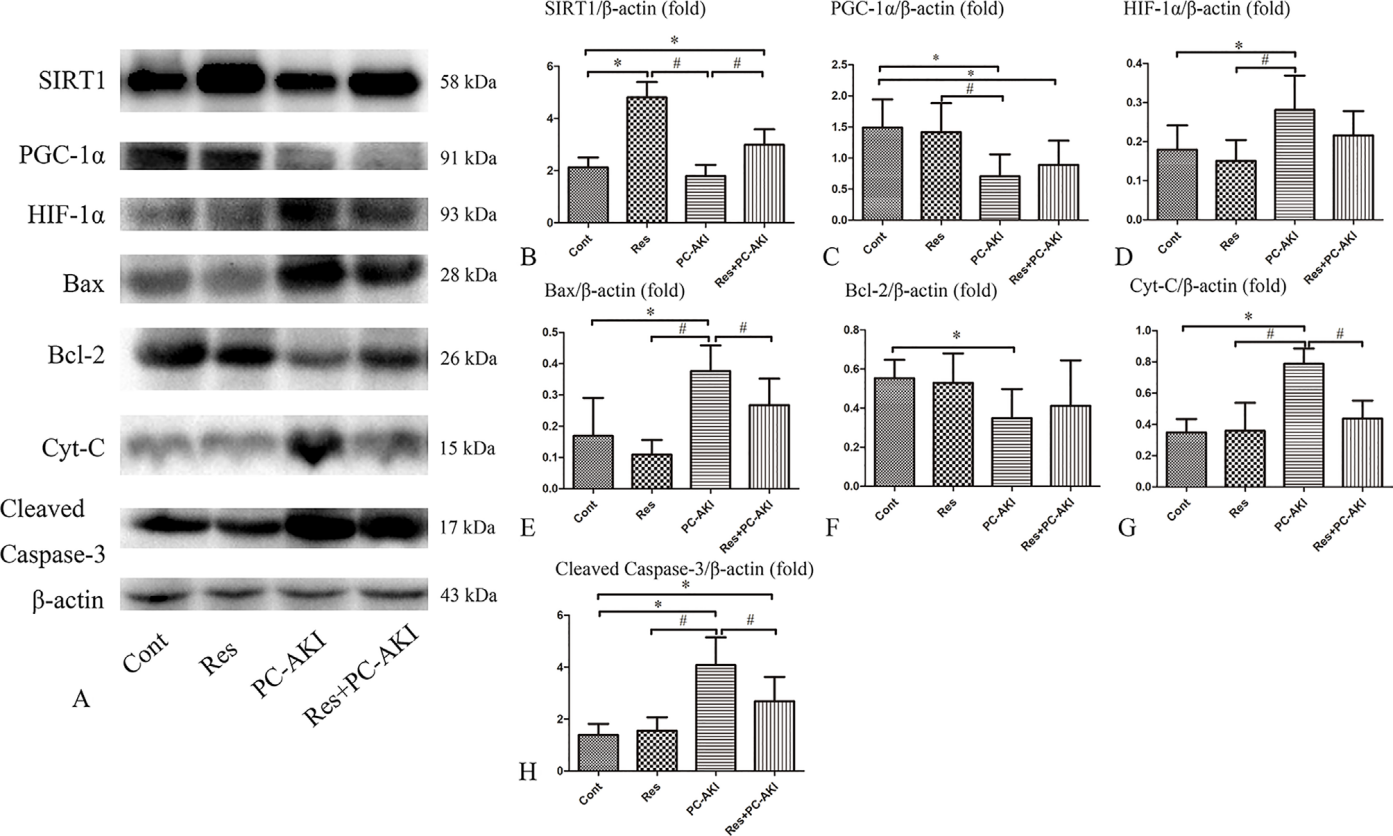
Effects of resveratrol on the expression of SIRT1, PGC-1α, HIF-1α and apoptosis proteins *in vivo*. **(A)** Activation of the SIRT1–PGC-1α–HIF-1α signaling pathway in PC–AKI associated with DN in rabbits. Representative western blot images of proteins in the rabbits with DN treated with saline (Cont), resveratrol alone (Res), iohexol (PC–AKI), and co-treatment with resveratrol and iohexol (Res+PC–AKI). **(B**–**H)** Relative densitometry analysis of the ratios of SIRT1–PGC-1α–HIF-1α signaling proteins to β-actin were expressed as mean ± standard error. *P < 0.05 vs. Cont; ^#^ *P* < 0.05 vs. PC–AKI. Cont, control; Res, resveratrol; PC–AKI, post-contrast acute kidney injury; SIRT1, silent information regulator l; HIF-1α, hypoxia-inducible transcription factor-1α; DN, diabetic nephropathy; PGC-1α, peroxisome proliferator-activated receptor gamma coactivator-1 alpha.

To investigate the molecular mechanisms associated with the renoprotective effects of resveratrol in DN rabbits with PC–AKI, the expression levels of SIRT1–PGC-1α–HIF-1α signaling proteins and those in its downstream pathway were analyzed (**Figure 7**).

Briefly, significantly higher levels of SIRT1 protein were detected in resveratrol-treated DN rabbits compared with rabbits in the control group [*P* < 0.001 (Res), *P* = 0.008 (Res+PC–AKI), respectively]. In addition, we found that the PGC-1α protein level was higher (*P* = 0.447) and the HIF-1α protein level lower (*P* = 0.112) in the Res+PC–AKI group compared to the PC–AKI group, but no statistical differences were observed between these two groups. Furthermore, the protein level of Bcl-2 (*P* = 0.515) was slightly increased, and those of Bax (*P* = 0.045), cleaved caspase-3 (*P* = 0.006) and Cyt-C (*P* < 0.001) were significantly lower in the Res+PC–AKI group compared with the PC–AKI group. In summary, resveratrol modulated SIRT1–PGC-1α–HIF-1α signaling proteins and associated pathways in DN rabbits by upregulating SIRT1 and PGC-1α and by downregulating HIF-1α, Bax, cleaved caspase-3 and Cyt-C protein levels.

### Effects of Resveratrol on SIRT1–PGC-1α–HIF-1α Signaling Under HG Conditions *In Vitro*

To further verify that SIRT1–PGC-1α–HIF-1α signaling was involved in renoprotection against PC–AKI under HG conditions, Ex527 and 2-MeOE2 were applied to HK-2 cells.

**Figure 8:**
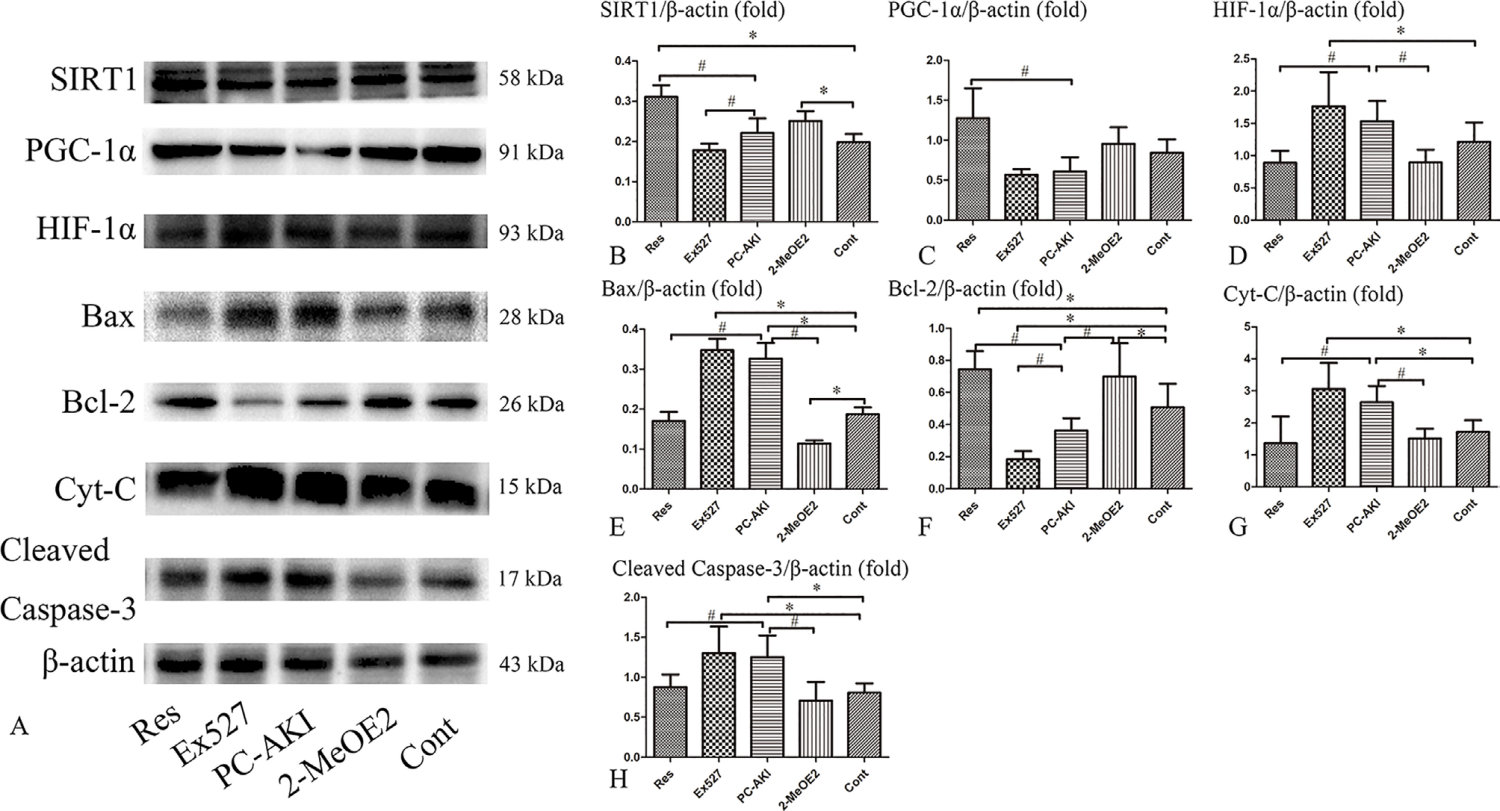
Effects of resveratrol on the expression of SIRT1, PGC-1α, HIF-1α and apoptosis proteins *in vitro*. **(A)** The expression of SIRT1, PGC-1α, and Bcl-2 proteins were lower in iohexol treated HK-2 cells compared with control cells, which was reversed by treatment with resveratrol. The expression of HIF-1α, Bax, cleaved caspase-3, and Cyt-C were markedly increased after iohexol injection, which was reversed by treatment with resveratrol. Furthermore, this effect was enhanced by HIF-1α inhibitor, 2-MeOE2. **(B**–**H)** Relative densitometry of SIRT1–PGC-1α–HIF-1α signaling proteins expressed as mean ± standard error. *P < 0.05 vs. Cont; ^#^ *P* < 0.05 vs. PC–AKI. Cont, control; Res, resveratrol; PC–AKI, post-contrast acute kidney injury; SIRT1, silent information regulator l; HIF-1α, hypoxia-inducible transcription factor-1α; Cyt-C, cytochrome C; PGC-1α, peroxisome proliferator-activated receptor gamma coactivator-1 alpha; Ex527, SIRT1 specific inhibitor; LG, low glucose. Groups: Res, HG+res+iohexol; Ex527, HG+Ex527+iohexol; PC–AKI, HG+iohexol; 2-MeOE2, HG+res+2-MeOE2+iohexol; Cont, LG.

As shown in **Figure 8**, SIRT1 and PGC-1α protein levels were increased after treating cells with resveratrol. Resveratrol significantly reduced the protein levels of HIF-1α and its downstream factors, namely cleaved caspase-3, Bax and cytochrome C; co-treatment with 2-MeOE2 enhanced this reduction under HG conditions. However, the protective role of the SIRT1–PGC-1α–HIF-1α pathway was suppressed by the SIRT1 inhibitor, Ex527. The expression of SIRT1 (*P* = 0.201), PGC-1α (*P* = 0.258), and Bcl-2 (*P* < 0.001) proteins in cells treated with iohexol and Ex527 were decreased and the expression of HIF-1α (*P* = 0.008), Bax (*P* < 0.001), cleaved caspase-3 (*P* = 0.001), and cytochrome C (*P* = 0.001) were significantly increased compared to the control group. In summary, resveratrol also modulated SIRT1–PGC-1α–HIF-1α signaling proteins and associated pathways in HK-2 cells under HG by upregulating SIRT1 and PGC-1α, and by downregulating HIF-1α, Bax, cleaved caspase-3 and cytochrome C protein levels in a SIRT1-mediated manner.

## Discussion

Post-contrast acute kidney injury is one of the leading causes of hospital-acquired acute kidney injury (Takahashi et al., 2017; Van Der Molen et al., 2018). Epidemiological and clinical evidence suggests that diabetes mellitus plays a part in the development of PC–AKI after the use of contrast medium (Tziakas et al., 2014; Chalikias et al., 2016). The identification of new molecular target(s) is thus crucial for the development of therapies for the prevention of PC–AKI in high-risk patients.

The development of PC–AKI with DN was found to lead to a decrease in the expression of SIRT1. Furthermore, we found that resveratrol could reverse the decrease in SIRT1 expression induced by PC–AKI with DN, which suggests that this may improve renal function, alleviate renal injury, and decrease iohexol-induced hypoxia and apoptosis in renal tubular cells. In addition, our findings also suggested that resveratrol may reduce iohexol-induced HK-2 cell apoptosis under HG conditions. Notably, resveratrol prevented the down-regulation of PGC-1α and up-regulation of HIF-1α in response to iohexol and DN in both a rabbit kidney and tissue culture model. These results indicate that resveratrol is an important survival mediator that protects against PC–AKI based DN complications by activating the SIRT1–PGC-1α–HIF-1α signaling pathway.

We found that resveratrol can protect the kidneys against damage induced by contrast medium in rabbits with DN. Suggested mechanisms for the protective action of resveratrol on PC–AKI with DN based on observations by us and others are as follows: 1) We found that resveratrol alleviates impaired renal hypoxia observed in PC–AKI as shown by observing functional MRI *R2* ^*^ parameters. 2) Resveratrol exerts a protective effect against contrast medium–induced injury by reducing oxidative stress and the excessive generation of reactive oxygen species (ROS) (Hussien et al., 2018). As reported, ROS may exert direct tubular and vascular endothelial injury, and further intensify renal parenchymal hypoxia by virtue of endothelial dysfunction and dysregulation of tubular transport (Andreucci et al., 2014). 3) We also found that resveratrol significantly alleviates tubular apoptosis in a PC–AKI animal model with DN.

PGC-1α has been characterized as a master regulator of mitochondrial biogenesis, which facilitates the combustion of stored energy by increasing its mitochondrial production (Dong et al., 2015; Ishimoto and Inagi, 2016). The deacetylation of PGC-1α ameliorates renal ischemic–reperfusion injury by recovering mitochondrial homeostasis (Funk and Schnellmann, 2013). As previously noted by Humes et al., contrast medium can impair mitochondrial energetics, resulting in a reduction in tubular ATP (Zager et al., 2003). We found that resveratrol countered the effect of contrast medium on mitochondrial function by increasing PGC-1α expression and therefore exerting a protective effect.

Previous studies have indicated that HIF-1α is upregulated in PC–AKI (Rosenberger et al., 2005). Accumulating evidence has also suggested that hypoxia has an important role in the development of PC–AKI (Wybraniec et al., 2015). In addition, diabetes mellitus with endothelial dysfunction may render the kidneys susceptible to further hypoxia by contrast medium (Calvin et al., 2010). Furthermore, hypoxia contributes to the formation of ROS and oxidative stress (Heyman et al., 2008). Similarly, contrast media can enhance oxidative stress leading to renal tubular epithelial cell apoptosis. In turn, that this may cause tissue hypoperfusion and hypoxia, which can aggravate the initial tissue damage caused by contrast medium, leading to a vicious circle (Liu et al., 2014; Patschan and Muller, 2016). It should be noted that resveratrol participates in PC–AKI with DN *via* a complex signaling network that includes PGC-1α, HIF-1α and other proteins, and that the inhibition of HIF-1α observed in our present study may be a dependent pathway through which resveratrol exerts its renal protective effects. Moreover, after treating cells with an inhibitor of HIF-1α, 2-MeOE2, the beneficial effect of resveratrol was greatly reinforced. Alternatively, given that SIRT1 expression and activity gradually decrease during hypoxia, HIF-1α may take over hypoxic signaling. In contrast to our results, Manotham et al. have suggested that HIF-1α has a role in a natural hypoxic adaptation to kidney ischemia that protects the medulla against ischemic insults (Manotham et al., 2005). Additionally, the potential protective impact of HIF upregulation has been extensively studied in AKI in a preclinical study (Loeffler and Wolf, 2015). Together with the discordant role of HIF-1α in AKI, we interpret these findings to mean that a “window of opportunity” for a HIF-dependent transcriptional response may exist. When the severity of hypoxia exceeds this, HIF-1α may begin to play a damaging role, resulting in the failure of this response and injury that may become irreversible.

In the present study, the elevated *D* observed in the renal cortex and medulla may be attributed to iohexol-induced tubular damage, renal tissue edema and inﬂammatory cell infiltration that may become alleviated after intervention with resveratrol. Meanwhile, we hypothesized that an increase in blood flow was responsible for increased D* and *f* values observed in the kidneys of resveratrol-treated rabbits; resveratrol, as an activator of SIRT1, may significantly decrease renal vascular dysfunction. Moreover, the application of iohexol led to a prolonged increase of R2* values and HIF-1α expression, which suggested that hypoxia may be a major factor associated with the early development of PC–AKI and was consistent with other studies (Li et al., 2015). The decrease in *R2* ^*^ values may be attributed to the inhibition of hypoxia production induced by resveratrol during the diabetes stage, and the amplification of secondary hypoxia signaling after iohexol injection. However, since there was very little blood flow change (the D*) in Res+PC–AKI rabbits compared with PC–AKI rabbits, we think that resveratrol may have led to a decrease in oxygen consumption to ameliorate renal glomerular hyperfiltration. Regardless of the mechanism, our findings support the concept that resveratrol may have decreased the iohexol-induced renal hypoxia observed. Therefore, in this manner, resveratrol may promote the recovery of renal function after contrast injury in DN.

Nevertheless, potential limitations exist in the present study that require discussion. 1) The PC–AKI model we used was based on a DN procedure that increased the complexity of the model. 2) The sample size was small, and the SIRT1 inhibitor was not used in rabbits. Thus the current *in vivo* study could not adequately confirm the effect of resveratrol and subsequent apoptosis as observed in renal tubular cells. 3) We only explored the effects of resveratrol on HK-2 cells; a reduction in SIRT1 activity may also occur in other types of kidney cells and this should be addressed in future studies. 4) The mitochondrial membrane potential was not measured in our study so that the nature of the apoptosis and degree of mitochondrial dysfunction observed are yet to be elucidated.

In summary, our results suggest that resveratrol may prevent the development of post-contrast acute kidney injury. Increased SIRT1–PGC-1α–HIF-1α signaling is associated with renoprotection against PC–AKI with DN. Such beneficial effects are mainly mediated *via* anti-hypoxia, anti-apoptotic and energy maintenance effects. Consequently, understanding the relationship between resveratrol and PC–AKI following diabetic nephropathy may lead to a greater clinical perspective on tackling this disease.

## Data Availability

The raw data supporting the conclusions of this manuscript will be made available by the authors, without undue reservation, to any qualified researcher.

## Ethics Statement

All animal studies (including the rabbit euthanasia procedure) were done in compliance with the regulations and guidelines of our university institutional animal care and conducted according to the AAALAC and the IACUC guidelines.

## Author Contributions

Conceptualization: KR, YW; Project Administration: YW, XQ, XZ; Methodology: BW; Data Curation: BW; Writing the Original Draft: YW; Writing, Review and Editing: KR, BW.

## Funding

The National Natural Science Foundation of China (Grant 81571635) and Scientific Research Foundation for Advanced Talents, Xiang’an Hospital of Xiamen University (NO. PM201809170011) supported this study.

## Conflict of Interest Statement

The authors declare that the research was conducted in the absence of any commercial or financial relationships that could be construed as a potential conflict of interest.

## Abbreviations

Res, Resveratrol; SIRT1, Silent information regulator l; PC–AKI, Post-contrast acute kidney injury; DN, Diabetic nephropathy; PGC-1α, Peroxisome proliferator-activated receptor gamma coactivator-1 alpha; HIF-1α, Hypoxia-inducible transcription factor-1α; HK-2, Renal tubular epithelial cells; 2-MeOE2, 2-Methoxyestradiol; Cr, Serum creatinine; BUN, Urea nitrogen; CysC, Cystatin C; uNGAL, Urinary neutrophil gelatinase–associated lipocalin; fMRI, Functional magnetic resonance imaging; BOLD, Blood-oxygenation-level–dependent; R2*, Apparent relaxation rate; IVIM, Intravoxel incoherent motion; *D*, Pure molecular diffusion; D*, Flow velocity; *f*, The fraction of water flowing in capillaries; HG, High glucose; ROS, Reactive oxygen species; Cyt-C, Cytochrome C.

## Supplementary Material

Figure S1 Experimental flow chart for the in vivo model of contrast-induced kidney injury in DN.

Figure S2 Positions of typical regions of interest (ROI). **(A)** T2-weighted image. **(B)** IVIM parametric images of D (b = 0 s/mm^2^). CO, renal cortex; OM, outer medulla; IVIM, intravoxel incoherent motion; D, pure molecular diffusion.

Figure S3 Experimental flow chart for the in vitro model of contrast-induced kidney cell injury in DN.
