# Supplementary figures and images for: Resveratrol Protects Against Post-Contrast Acute Kidney Injury in Rabbits With Diabetic Nephropathy
Source: Front Pharmacol. 2019 Jul 26;10:833. doi: 10.3389/fphar.2019.00833 (PMC6675867; doi:10.3389/fphar.2019.00833)

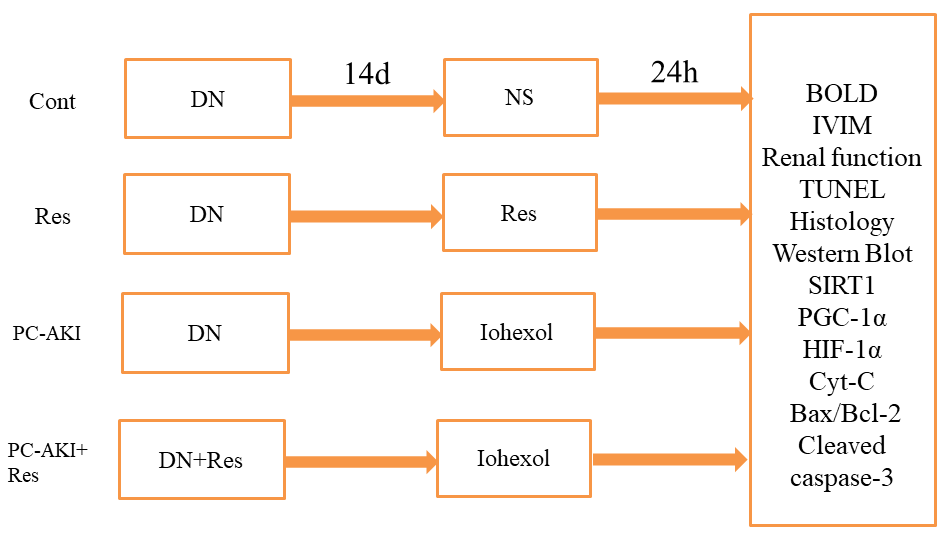

Supplement: Figure S1 — Experimental flow chart for the in vivo model of contrast-induced kidney injury in DN. [file Image_1.tif]

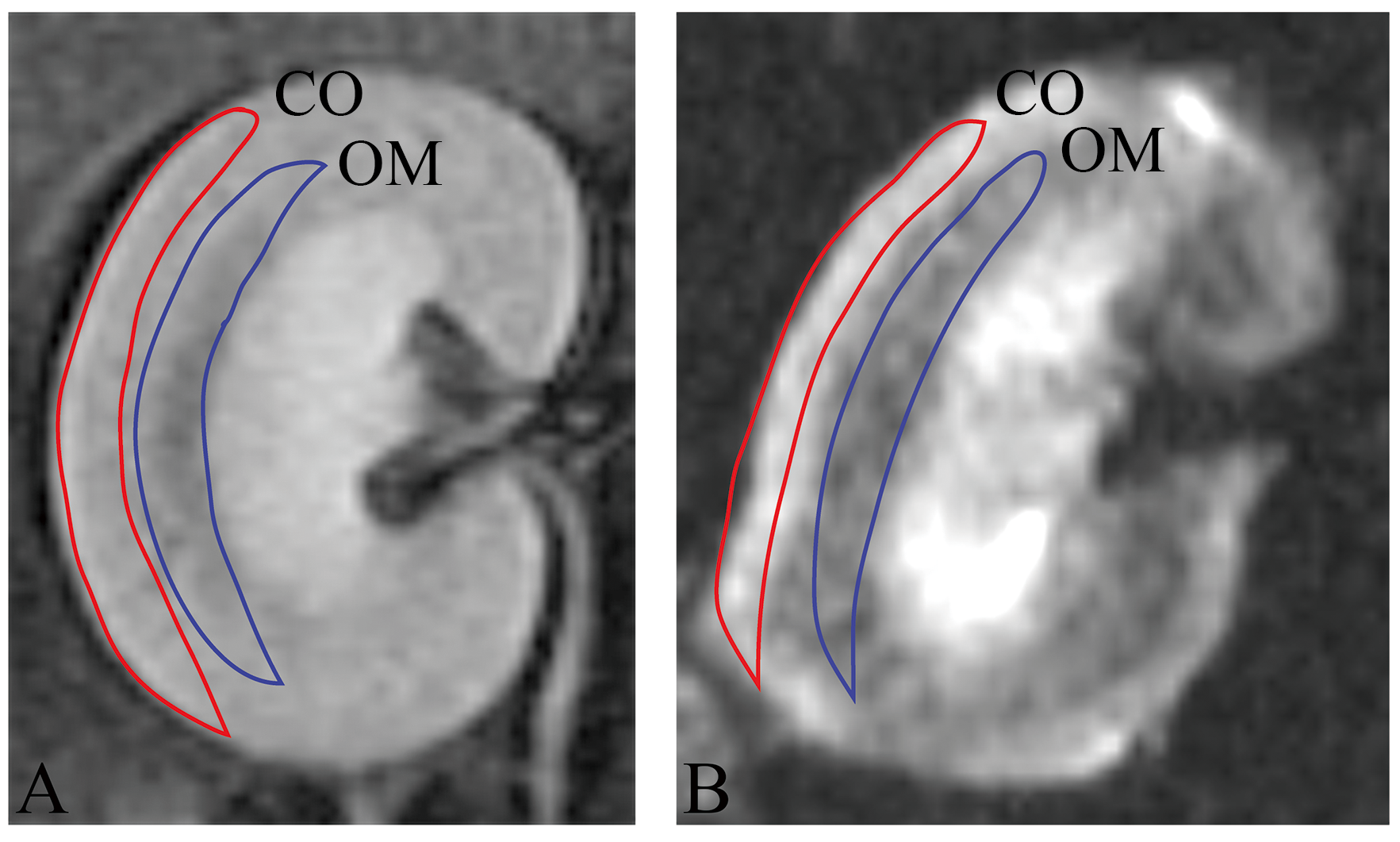

Supplement: Figure S2 — Positions of typical regions of interest (ROI). (A) T2-weighted image. (B) IVIM parametric images of D (b = 0 s/mm2). CO, renal cortex; OM, outer medulla; IVIM, intravoxel incoherent motion; D, pure molecular diffusion. [file Image_2.tif]

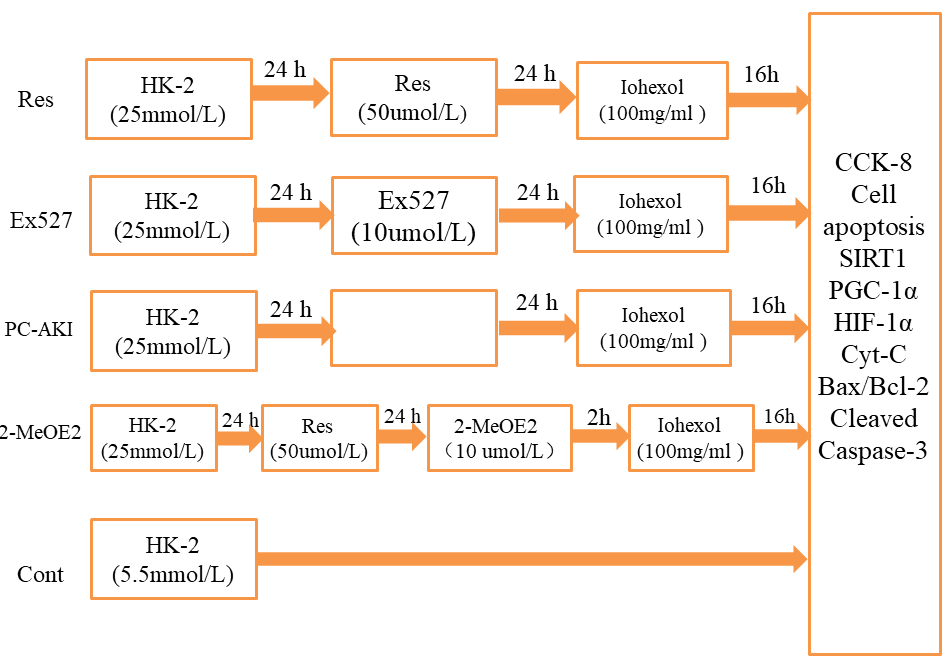

Supplement: Figure S3 — Experimental flow chart for the in vitro model of contrast-induced kidney cell injury in DN. [file Image_3.tif]
